# Capturing the value of vaccination: impact of vaccine-preventable disease on hospitalization

**DOI:** 10.1007/s40520-022-02110-2

**Published:** 2022-05-28

**Authors:** Mark T. Doherty, Emmanuel Aris, Nathalie Servotte, Ekkehard Beck

**Affiliations:** grid.425090.a 0000 0004 0468 9597 GSK, Building W23, 20 Avenue Fleming, 1300 Wavre, Belgium

**Keywords:** Vaccination, Complications, Burden of disease, Hospitalization

## Abstract

Evidence from epidemiological studies suggests that vaccine-preventable disease (VPD) such as influenza or herpes zoster contribute significantly to the increased risk of older adults for cardiovascular, cerebrovascular, neurological, and renal complications in the period after illnesses. However, since the period of elevated risk can persist well beyond the duration of the acute illness, the connection is not always recognized. To obtain insights into the relationship between diagnoses for vaccine-preventable disease and for other conditions, we analyzed principal and secondary diagnoses for 3,127,768 inpatient admissions of adults 50 years and older in the United States, using medical insurance claims drawn from the IBM^®^ MarketScan^®^ Research Databases (Marketscan). The Marketscan data indicated that overall, 3.1% of these hospitalizations had a principal diagnosis of VPD with variation by month of admission, and age. However, hospitalizations with a principal non-VPD diagnosis but secondary VPD diagnoses were 2.8 times more frequent, with particularly high rates in those whose principal diagnoses were non-VPD respiratory or circulatory disease. Hospitalized patients with a secondary VPD diagnosis tended to have poorer discharge outcomes, and longer length of stay in comparison to hospitalized patients without a secondary VPD diagnosis. In total, these data are consistent with suggestions that VPDs play a significant and potentially under-estimated role in hospitalization and outcomes, which may be potentially preventable by improved vaccination coverage.

**Supplementary Information:**

The online version contains supplementary material available at 10.1007/s40520-022-02110-2.

## Introduction

While the stress placed on society by the coronavirus disease 2019 (COVID-19) pandemic–ranging from reduced capacity of healthcare facilities to deal with other illnesses, to economic carnage [1, 2]—has attracted headlines, it is less appreciated that the susceptibility of older adults to infection [3] is, in effect, a “slow, silent pandemic” with many of the same characteristics. Globally, aging populations are already accelerating demand for primary care, placing healthcare systems and budgets under growing strain. To take a single example, emergency admissions increased in England and Wales by 42% over the period 2006/07 to 2015/16 at an estimated additional cost of £5.5 billion, with the greatest increase (58.9%) in those over 85 [4]. Data from Italy in the period 1995–2011 indicate that the profile of patients admitted to infectious disease wards was skewing toward older, polymorbid individuals [5], while in the United States between 2003 and 2013, secondary diagnoses for infectious diseases rose from 7.8 to 15.1% in 58.9% in patients admitted to intensive cardiac wards [6].

The burden on healthcare services due to infectious disease is further exacerbated by the seasonal nature of many of these infections (for example, influenza) leading to pressure on healthcare facilities which can exceed capacity at times of peak demand. As an example, during the winter of 2017/18 in England, on average, 20 hospital trusts reached occupancy levels of over 99% on any given day, leading to prolonged delays for admission, treatment and ambulance handover (and therefore ambulance availability) [7]. Delays of this kind can ripple across the entire spectrum of treatment, leading to generally poorer clinical outcomes, poorer patient experience, and a higher risk that patients will leave the facility without being seen. Pressure on the number of beds can lead to cancellation of selective treatments, placement on clinically inappropriate wards, premature discharge, and severe pressure on staff, all of which can increase the risk of errors, an increased chance of avoidable and costly hospital readmissions and (particularly in intensive care units) increased risk of mortality for patients [8–11].

The burden of infectious disease is thus not trivial, even in high-income countries: it has been estimated that just 4 vaccine-preventable diseases (VPDs)—influenza, pneumococcal disease, herpes zoster and pertussis cost the US $26.5 billion in direct medical and societal cost annually, with the largest part due to influenza [12]. This disease burden is only expected to grow in the immediate future, with the annual societal economic burden for the four commonest VPDs projected to increase from approximately $35 billion to $49 billion over the next 30 years [13, 14]. The aging of the patient population further exacerbates bed pressure due to VPDs as the median length of hospital stay for diseases like influenza increases with age [15].

Significant as these costs are, they are likely to be underestimates, as they only assess the costs of treating VPDs themselves and typically do not take into account the downstream effects that can arise as a result of infection. The best-described of these is the strong correlation between influenza infection and mortality from cardiovascular or cerebrovascular disease, initially identified by the strong temporal association. Subsequent analyses suggests that 35–50% of all excess deaths associated with seasonal epidemic influenza are due to cardiovascular disease [16–19], with the correlation particularly strong for acute myocardial infarction, probably due to destabilization of atherosclerotic plaques, leading to coronary artery occlusion, and driven by the impact of influenza infection on inflammatory and coagulation mechanisms [20]. A number of studies have also highlighted an increased risk of transient ischemic stroke and myocardial infarction following acute infection with, or reactivation of, varicella zoster virus and as with influenza, the mechanism has been linked to infection-driven vascular changes and proinflammatory conditions that can result in multiple serious downstream effects [21–24]. The observation that vaccination against these infections can reduce the risk of cardiovascular or cerebrovascular mortality also provides strong support for a causal role of the infections [25, 26], but the finding that risk can in some cases remain elevated for more than a year after the acute infectious episode [27], means that the relationship between the illness which leads to hospital admission and the infection which may have contributed to it, may often be missed.

As a first step towards developing more accurate estimates of the costs associated with VPD in adults in the inpatient setting, we analyzed diagnoses drawn from insurance claims records from the IBM^®^ MarketScan^®^ (Marketscan) Commercial Claims and Encounters, Medicare Supplemental, and Medicaid databases from the period 01 July 2016–30 June 2019, and stratified by month of admission, principal and secondary diagnoses, length of stay (LoS) and discharge/outcome. For the purposes of this study, the focus has been on older adults (defined as those 50 years of age and older), based on their increasing risk of illness from infectious disease, including VPDs [28]. This is also the segment of the population that is growing proportionately at the fastest rate, and for whom aggregate healthcare costs are growing the fastest. The Centers for Disease Control and Prevention (CDC) in the United States recommends vaccination for VPDs such as herpes zoster vaccine for adults from 50 years onwards, but mitigation is typically poor, with most countries reporting vaccination coverage at far lower levels than for equivalent pediatric vaccines [29, 30]. The data presented here allow some insight into the magnitude and specifics of VPD burden in this older adult inpatient population and will hopefully help to inform more detailed prospective studies at the hospital and primary healthcare level.

## Methodology

### Study design

This retrospective cohort study utilized anonymized medical claims records from the MarketScan Commercial Claims and Encounters (Commercial), Medicare Supplemental (Medicare) and Multistate Medicaid (Medicaid) databases from July 1, 2016 to June 30, 2019. The MarketScan Commercial contains medical and drug data of over 42 million lives between 2017 and 2019 (employees, their spouses, and dependents) who are covered by employer-sponsored private health insurance in the US. The MarketScan Medicare database contains medical and drug data of around 2 million lives between 2017 and 2019 with Medicare supplemental insurance paid by employers. The MarketScan Medicaid database contains data of around 18 million lives between 2017 and 2019 from Medicaid enrollees from multiple states [31].

The MarketScan data used for this study contained de-identified patient-level health care claims related to inpatient encounters at academic and non-academic inpatient facilities across the US, and included claims from patients with both private and federal health insurance. Medical records include diagnoses using the International Classification of Diseases, 10th Revision (ICD-10) codes, vaccinations, medication fills, laboratory results, vital signs, the type of healthcare resource used and demographic data [32]. Classification of infectious diseases as either VPD or non-VPD was based on the World Health Organization (WHO) VPD classification as of October 2020 [33].

Detailed variable definitions are shown in Table 1. This study was approved by an internal review board as only de-identified data were used.

**Table 1:** Frequency of principal diagnoses during hospitalization restricted to principal non-Vaccine Preventable Disease followed by a secondary Vaccine Preventable Disease diagnosis (Commercial/Medicare)

ICD-10 code	Diagnoses	*n* (%)^a^ *N* = 148,738
A419	Sepsis, unspecified organism	37,626 (25.30)
J440	Chronic obstructive pulmonary disease with (acute) lower respiratory infection	6839 (4.60)
J441	Chronic obstructive pulmonary disease with (acute) exacerbation	5376 (3.61)
J690	Pneumonitis due to inhalation of food and vomit	4695 (3.16)
J9601	Acute respiratory failure with hypoxia	4002 (2.69)
I110	Hypertensive heart disease with heart failure	3509 (2.36)
I130	Hypertensive heart and chronic kidney disease with heart failure and stage 1 through stage 4 chronic kidney disease, or unspecified chronic kidney disease	3362 (2.26)
J9621	Acute and chronic respiratory failure with hypoxia	3043 (2.05)
I214	Non-ST elevation (NSTEMI) myocardial infarction	2336 (1.57)
N179	Acute kidney failure, unspecified	2114 (1.42)

*ICD-10* International Classification of Diseases tenth revision

^a^% = (*n*/*N*) × 100, *N* = number of total diagnoses, and *n* = number of diagnoses in a given category

The goal of the study was hypothesis-generating: to determine to what degree, and to what extent, VPD diagnoses captured as principal and secondary diagnoses could be tracked using a claims database. For this reason, although COVID-19 infection is a VPD and appears to also be associated with elevated downstream risk for cardiovascular disease [34], COVID-19 is not covered in the current study. Data collection was specifically limited to 2016–2019 to (a) prevent potential bias from a pandemic setting, which may limit generalizability, and (b) to ensure compatibility of diagnostic coding across the period of analysis. COVID-19 can, however, reasonably be expected to contribute further to the burden of hospitalization in older adults, even when we discount severe disease arising from acute infection [35], so will need to be taken into account in subsequent studies.

Discharge status was defined from the status of patient upon discharge information contained in the hospitalization summary claim, and has been recategorized into seven broad categories (see Supplementary figures).

### Eligibility and cohort selection

Only male and female individuals of at least 50 years of age were considered. A cut-off of 50 years of age was chosen to align with CDC recommendations for older adult vaccination in the US. These subjects must all have had at least one hospitalization between July 1, 2016 and June 30, 2019. All participants who fulfilled these criteria were included in the analysis. Enrollees of MarketScan Commercial of Medicare were pooled together for analysis purposes. These populations are often considered to come from the same pool of individuals, as Medicare advantage coverage beneficiaries were typically previously enrolled in a commercially insured plan. In contrast, beneficiaries in the MarketScan Medicaid database represent a different type of population (subjects receiving state aid) and were, therefore, analyzed and presented separately.

### Assignation of patient diagnoses

Hospitalizations were classified according to the broad ICD-10 code range of their principal diagnosis (diagnosis constituting the main reason for an admission, usually the “discharge” diagnosis), and secondary diagnoses. The distribution of the frequency of the different code ranges were presented, along with the percentage of hospitalizations with VPD as primary diagnosis stratified by month of admission, of by age group of the individual (50–64, 65–79 and ≥ 80 years of age). Similarly, distribution of primary non-VPD diagnosis, but with at least one secondary diagnosis of VPD or not were also tabulated. The lack of information on patient background and origin in the anonymized data precluded any formal analysis of incidence or disease-associated risk associated with admission.

Burden of VPDs in hospitals assessed by secondary diagnosis was evaluated by estimating the average length of stay of a hospitalization with a secondary VPD diagnosis to another hospitalization with the same principal non-VPD diagnosis, age group, and epidemiological year.

## Results

### Burden of VPDs in inpatients assessed by principal diagnosis captured in Marketscan databases

**Fig. 1:**
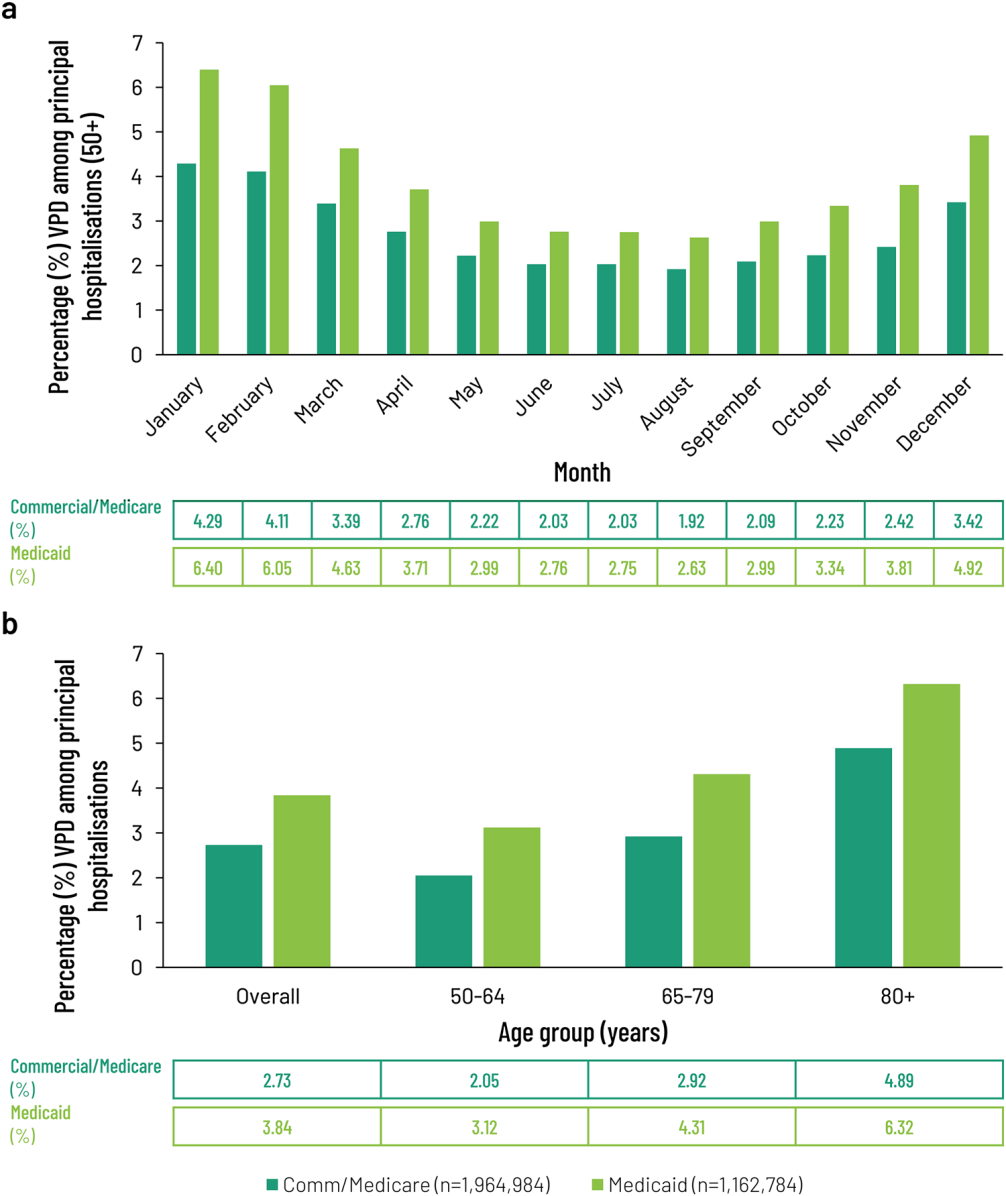
Distribution of patients with a principal diagnosis of VPD at admission, stratified according to the month that the claim was registered (**a**) or the age of the patient at the time of admission (**b**) and expressed as a percentage of all claims registered with the MarketScan Commercial Claims and Encounters (Commercial), Medicare Supplemental (Medicare) and Multistate Medicaid (Medicaid) databases registered by month or age from July 1, 2016 to June 30, 2019. Data from Commercial/Medicare were pooled (*n* = 1,964,984) according to standard practice and presented alongside Medicaid claims (*n* = 1,162,784). *VPD* vaccine-preventable diseases

Overall, 3,127,768 inpatient admissions of adults 50 years and older were identified and considered in the analyses. Of these, 1,964,984 were classified as Commercial/Medicare and 1,162,784 as Medicaid. The initial analysis of the principal diagnoses recorded by broad category (see Supplementary Material, Fig. S1) showed that VPDs constituted a relatively minor proportion of all diagnoses (3.14%—11th most common principal diagnosis on admission). Diseases of the circulatory (19.92%), musculoskeletal (14.92%) and digestive (11.77%) systems were the most common. Injury, poisoning and external causes (8.57%) were the fourth most common principal diagnosis. Principal diagnoses for VPD showed evidence of seasonality (Fig. 1a), with a winter peak centered on January, which at 4.29% of all Commercial/Medicare admissions was more than twice the level seen at the trough (August, 1.92%). Admissions for the Medicaid cohort displayed a similar seasonal pattern (Fig. 1a), consistent with previous reports [36]. There was also an apparent difference when the data on principal VPD diagnoses were stratified by age (Fig. 1b) with principal diagnoses for VPDs making up 4.89% of all Commercial/Medicare admissions for those 80 + years of age, versus 2.05% for those in the age range 50–64 years.

A principal diagnosis for VPD ranged from 2.73 to 3.84% of annual admissions in patients 50 + years of age claiming under Commercial/Medicare and Medicaid, respectively, with similar outcomes seen whether stratified by month of admission (Fig. 1a) or age (Fig. 1b). The seasonal peak of admissions reached 6.40% for patients claiming via Medicaid and those 4.29% for those with Commercial or Medicare coverage (Fig. 1a). Trend analysis for month of admission showed that admissions with a principal diagnosis of VPD ascribed to either insurance category tracked closely with a second order polynomial curve (*R*^2^ = 0.9548 for Commercial/Medicare admissions, *R*^2^ = 0.967 for Medicaid admissions). Admissions for all causes did not show such marked seasonality (*R*^2^ = 0.6669).

### Burden of VPDs in hospitals associated with both principal and secondary diagnoses captured in Marketscan databases

**Fig. 2:**
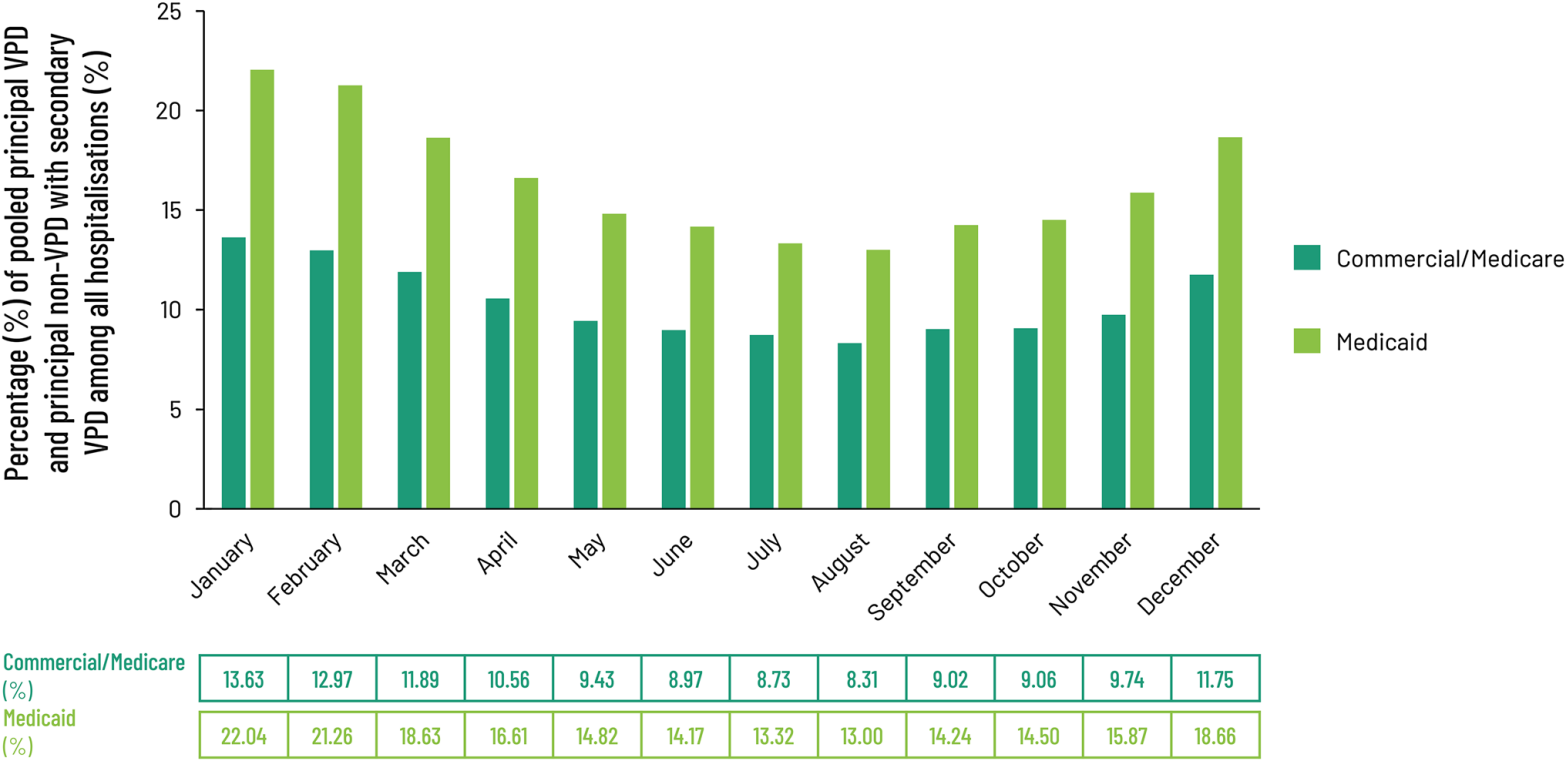
Distribution of all patients with a diagnosis of VPD, either as a principal or secondary diagnosis, stratified according to the month that the claim was registered and expressed as a percentage of all claims registered with the MarketScan Commercial Claims and Encounters (Commercial), Medicare Supplemental (Medicare) and Multistate Medicaid (Medicaid) databases registered by month or age from July 1, 2016 to June 30, 2019. Data from Commercial/Medicare were pooled (*n* = 1,964,984) according to standard practice and presented alongside Medicaid claims (*n* = 1,162,784). *VPD* vaccine-preventable diseases

We also analyzed the overall proportion of admissions with concurrent VPD infections among Commercial/Medicare patients by pooling those with either a principal diagnosis of VPD or a principal non-VPD diagnosis, but with at least one secondary diagnosis of VPD. Admissions with a principal non-VPD diagnosis, but with at least one secondary diagnosis of VPD made up a higher percentage of all admissions than those with a principal diagnosis of VPD (Figs. 1a, 2). In the Commercial/Medicare cohort, VPD as a secondary diagnosis was 290% more frequent than a principal VPD diagnosis in the 50–64 and 65–79 age groups while a 240% difference was seen in patients 80 + at admission. Using this approach, a similar seasonal pattern of admissions was seen for admissions with either a principal or secondary diagnosis of VPD, both showing a December-January peak and a June–August nadir, but the overall burden of disease associated with a principal non-VPD diagnoses with at least one secondary diagnosis of VPD was substantially larger than that for principal VPD diagnoses alone throughout the year (Figs. 1a, 2). Stratifying these data by claim type (Commercial/Medicare versus Medicaid) showed a similar pattern of seasonality in both groups (Fig. 2). As for principal VPD diagnosis, trend analysis showed that admissions with a principal diagnosis of non-VPD and a secondary diagnosis of VPD ascribed to either insurance category tracked closely with a second order polynomial curve (*R*^2^ = 0.9634 for Commercial/Medicare admissions, *R*^2^ = 0.9661 for Medicaid admissions). As seen for admissions with a principal diagnosis of VPD alone, admissions among Medicaid patients for pooled principal and secondary VPD diagnoses showed both more exaggerated seasonality and represented a higher proportion of all admissions, than did admissions among Commercial/Medicare patients (Figs. 1a, 2) but in the absence of incidence data for the communities from which these populations are drawn, it was not possible to make any analysis on whether these differences are meaningful.

### Burden of VPDs in hospitals assessed by secondary diagnosis

To gain some insight into the principal diagnoses in patients with a secondary diagnosis of VPD, we analyzed the distribution of specific principal diagnoses on admission for non-VPD conditions followed by a secondary diagnosis of VPD. Secondary diagnoses of VPD are unequally distributed among the hospitalizations with non-VPD principal diagnoses: with 70.3% of the secondary diagnoses for VPD being associated with just 3 non-VPD principal diagnoses, which themselves only constitute 31.8% of the total admissions (see Supplementary Material, Fig. S1, Fig. S2) for Commercial/Medicare patients. These diagnoses are Certain infectious and parasitic diseases (ICD-10 Code range A00-B99), Diseases of the respiratory system (ICD-10 code range J00–J99) and Diseases of the circulatory system (ICD-10 code range I00–I99). A similar pattern was found among Medicaid patients with these same three diagnoses (ICD-10 Code ranges A00–B99, J00–J99 and I00–I99) making up 40.8% of total admissions (principal diagnoses), but accounting for 75.5% of all hospitalizations with non-VPD principal diagnoses and at least one secondary diagnosis of VPD. Secondary diagnosis of a VPD during hospitalization with a principal non-VPD diagnosis was made in 54.6% of Commercial/Medicare patients on admission, while 86.23% of the remaining diagnoses were made on day 1 of admission and 96% by day 2. Similar outcomes were reported for Medicaid patients, with 66.4% of secondary diagnoses for VPD being made at admission, and 94.5% by day 2 of admission. These data suggest that in almost all cases, the VPD infection diagnosed was acquired before hospitalization and was not acquired in the healthcare facility.

### Association of a secondary diagnosis of VPDs with outcomes

**Fig. 3:**
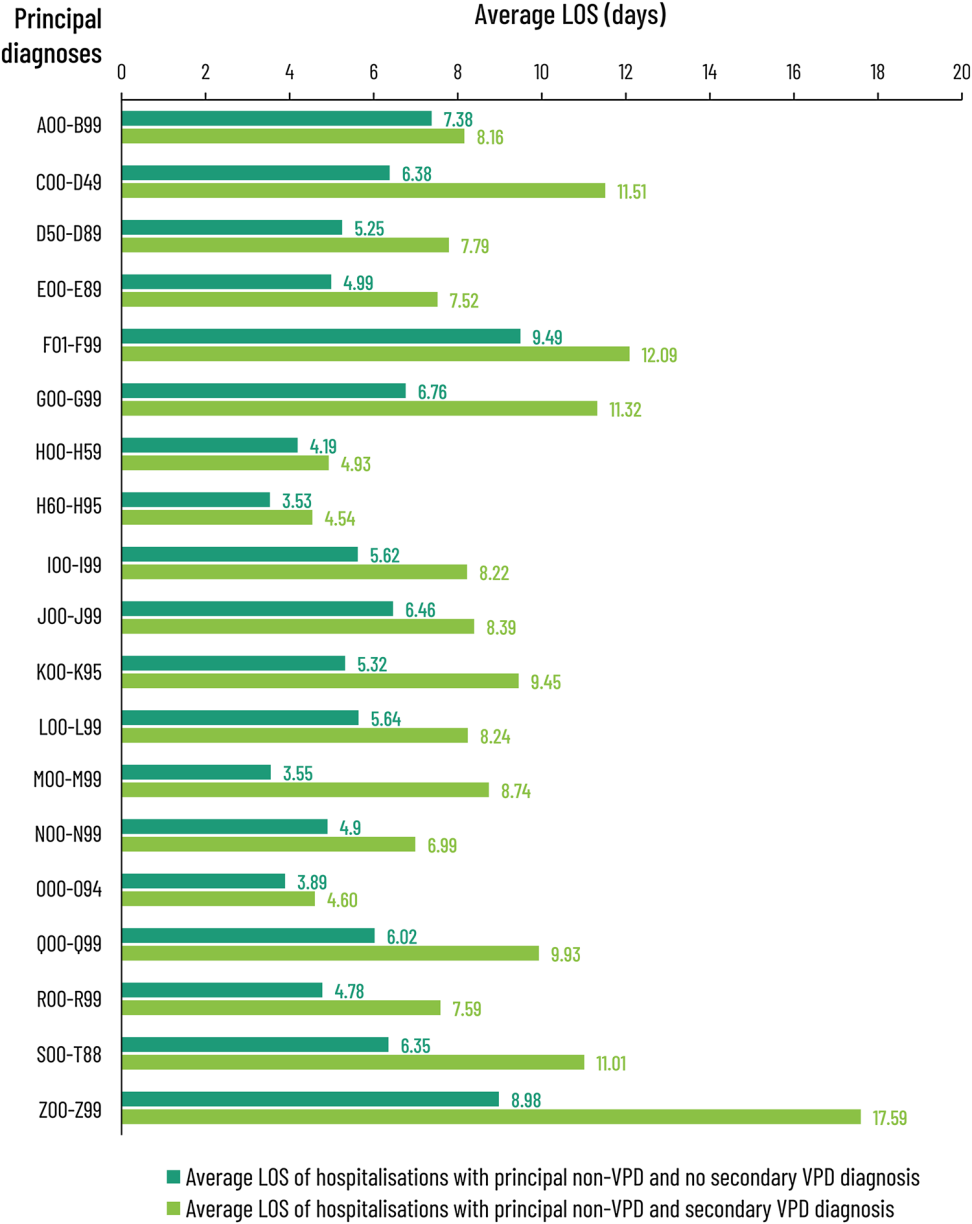
Length of stay in the initial facility where they were admitted (expressed as days, based on day of admission and discharge), for patients with a principal non-VPD diagnosis without secondary VPD compared to a principal non-VPD with secondary VPD. The data are drawn from all claims registered with the MarketScan Commercial Claims and Encounters (Commercial), Medicare Supplemental (Medicare) and Multistate Medicaid (Medicaid) databases from July 1, 2016 to June 30, 2019. Data from Commercial/Medicare were pooled (*n* = 1,964,984) according to standard practice and presented alongside Medicaid claims (*n* = 1,162,784). *VPD* vaccine-preventable diseases, *A00-B99* Certain infections and parasitic diseases, *C00-D49* Neoplasma, *D50–D89* diseases of the blood and blood-forming organs and certain disorders involving the immune mechanism, *E00–E89* endocrine, nutritional and metabolic diseases, *F01–F99* mental, behavioural and neurodevelopment disorders, *G00–G99* diseases of the nervous system, *H00–H59* diseases of the eye and adnexa, *H60–H95* diseases of the ear and mastoid process, *I00–J99* diseases of the circulatory system, *J00–J99* diseases of the respiratory system, *K00–K95* diseases of the digestive system, *L00–L99* diseases of the skin and subcutaneous tissue, *M00–M99* diseases of the musculoskeletal system and connective tissue, *N00–N99* diseases of the genitourinary system, *O00–O94* pregnancy, childbirth and puerperium, *Q00–Q99* congenital malformation, deformations and chromosomal abnormalities, *R00–R99* symptoms, signs and abnormal clinical and laboratory findings, not elsewhere classified, *S00–T88* injury, poisoning and certain other consequences of external causes, *Z00–Z99* factors influencing health status and contact with health services

Finally, we looked at the impact of a VPD diagnosis—as either a principal or a secondary diagnosis—on LoS and discharge. A comparison was done of the average LoS for Commercial/Medicare patients with a principal non-VPD diagnosis without secondary VPD versus those with the same principal non-VPD but with a secondary diagnosis of VPD. As shown in Fig. 3, having a secondary diagnosis for VPD increased the LoS for all patients with principal non-VPD diagnoses by 1.1- to 2.5-fold, leading to an average increase in LoS of 3 days. When stratified by age, the increase in LoS for Commercial/Medicare patients with a principal non-VPD diagnosis without secondary VPD to LoS for those with a principal non-VPD and secondary diagnosis of VPD was 4 days for those 50–64 years of age, 3 days for those 65–79, and 2 days for those 80 + . A similar average increase in LoS (3 days) was observed for Medicaid patients (data not shown), suggesting that outcomes were generally worse for patients with secondary VPD diagnoses, regardless of their principal non-VPD diagnosis or insurance status.

**Fig. 4:**
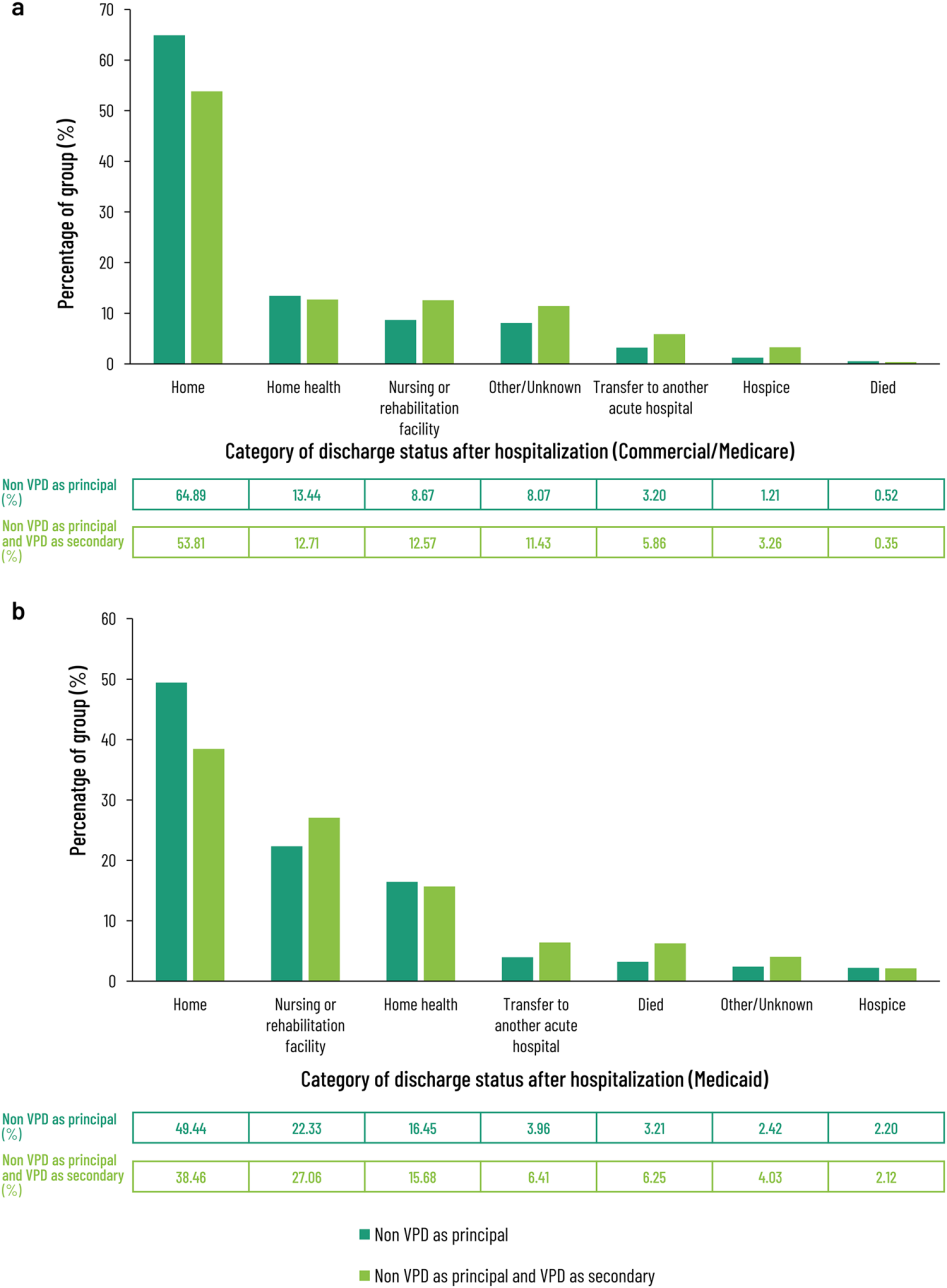
Category of recorded discharge status after hospitalization for patients with either a principal non-VPD diagnosis without secondary VPD compared to a principal non-VPD with secondary VPD for data drawn from all claims registered with the MarketScan Commercial Claims and Encounters (Commercial), Medicare Supplemental (Medicare) database: **a** (pooled *n* = 1,162,784), and the Multistate Medicaid (Medicaid) database: **b** (*n* = 1,162,784) from July 1, 2016 to June 30, 2019. *VPD* vaccine-preventable diseases

Discharge data also suggest worse outcomes for patients with secondary VPD diagnoses compared to those with the same principal diagnosis alone (Fig. 4). Among the Commercial/Medicare patients, with a principal non-VPD diagnosis, 65% and 13% were discharged to home or to home with home healthcare, respectively (Fig. 4). The corresponding figures for Commercial/Medicare patients with a principal non-VPD diagnosis and a secondary VPD diagnosis were 54% and 13%, respectively. Those patients with a principal non-VPD diagnosis and a secondary VPD diagnosis were more likely to be discharged to a nursing or rehabilitation facility, be transferred to another hospital, or to a hospice (Fig. 4a). The pattern for Medicaid recipients was similar, but not identical (Fig. 4b) for both those patients with a principal non-VPD diagnosis and those with a principal non-VPD diagnosis and a secondary VPD diagnosis. Only 49.4% of the former and 38.5% of the latter were discharged to home, while the remainder were discharged to a nursing or rehabilitation facility, another hospital, or died (Fig. 4b).

## Discussion

There are multiple observational and retrospective database studies which suggest that instances of some infectious diseases—including VPDs—are associated with a significantly elevated risk of subsequent illness or hospitalization for other, apparently unrelated conditions. This is by no means a new observation—the indirect mortality due to other infections subsequent to measles was first described by Thomas Sydenham in 1674 [37]. The immunodeficiency that develops in the wake of measles infection, and which can lead to substantial excess mortality in subsequent years due to non-measles infections is now well-described [28, 38]. Though these deaths are not due to measles per se, and are diagnosed as due to other causes, the fact that they can be prevented by vaccination against measles, and the description of the biological mechanism indicates a causal role for measles infection [28]. This linkage, where an infectious disease sets up the conditions that lead to increased risk of subsequent illness, but where the pathogen itself does not directly initiate the subsequently diagnosed condition is referred to as a “downstream effect” of the infection [16, 17].

A recent modelling analysis suggested that up to 8.4% of the disability and death attributed to non-communicable disease appears to be directly caused by prior infection, with a majority due to hepatitis B, streptococcal, and cervical cancer due to human papilloma virus—all VPDs [39]. This is a very conservative estimate, because the analysis only looked at diseases for which infection was the direct, proximal cause—even if diagnosed long after infection—and did not assess downstream effects. Published observational studies suggest a strong correlation between some VPDs and subsequently elevated risk of admission and mortality from non-communicable disease such as stroke and cardiovascular disease [16–19]. While the data from these studies suggest a correlation between some viral infections and subsequently elevated risk of hospital admission and mortality from non-communicable disease such as stroke and cardiovascular disease, there is little or no data on the proposed association of VPD and admissions for non-communicable diseases at the patient level.

We have, therefore, analyzed US medical claims records from the IBM Marketscan databases from over 3 million hospital admissions and studied the frequency of VPD as principal or secondary diagnoses on these hospitalizations. VPDs were observed in a substantially higher number of patients admitted for care than might be suggested by a principal diagnosis of VPD alone. Furthermore, secondary diagnoses of VPD were not equally distributed among the different classes of principal diagnoses, with admissions for circulatory and respiratory disease over-represented. While it is not possible to make any definitive claims in the absence of incidence data in the populations these patients were drawn from, this finding is consistent with previously published observational studies suggesting elevated risk for hospital admission or cardiac unit admission after some VPD infections [17, 21, 40]. It is also worth noting that almost all (95.3%) secondary diagnoses for VPDs in these cohorts were made within 24 h of admission. This strongly suggests that these VPDs had been acquired before admission, consistent with the hypothesis that, at least in some cases, they may have contributed to the admission and possibly to the increased LoS in those patients with a secondary diagnosis of VPD (Fig. 3).

In some cases, the connections between the principal non-VPD diagnosis and the secondary VPD diagnosis can be plausibly explained—an analysis of the specific principal diagnoses most commonly found in Commercial/Medicare patients with a secondary diagnosis of VPD (Table 1) shows that sepsis due to an unspecified organism was the most common, accounting for over a quarter of the diagnoses, with subsequent identification of the causative agent allowing assignation as a VPD. Similarly, the close temporal association of VPD secondarily diagnosed to the principal non-VPD diagnosis at admission, is consistent with a potential causal relationship between these events in at least some of the patients. For example, a principal diagnosis of chronic obstructive pulmonary disease (COPD) with acute lower respiratory infection, or with acute exacerbation (4.6% and 3.6% of principal diagnoses, respectively), is consistent with the extensive literature on COPD exacerbation that may be triggered by infections [29]. For other non-VPD principal diagnoses, however, such as myocardial infarct or heart failure, a potential causative role of the secondarily diagnosed VPD cannot be assumed, but is plausible, given the previously documented association [18, 41].

The data are, therefore, compatible with the hypothesis that community-acquired VPDs can potentially increase admissions and LoS to a greater extent than previously appreciated which may increase bed utilization and crowding at times of peak demand. Overlooking the impact of VPDs and their associated burden in the hospital setting could negatively impact the decision on promoting uptake of existing programs or whether to implement a vaccination program or not [42]. Prior observational studies indicate that vaccination programs can reduce the risk of hospital admissions in adults (particularly high-risk older adults, who typically have higher healthcare utilization rates) and the implicit suggestion from this study that it may be able to reduce in-hospital costs and resource utilization [43–46]. Adult vaccination remains underutilized, especially in older adults, so quantifying to what extent this may be possible will be of increasing importance in societies with aging populations.

It must be noted that the analyses presented here may suffer from several limitations. First, the principal diagnoses entered in the health care claim may not always indicate the disease that is causal of the hospitalization, and not all secondary diagnoses may have been recorded. Second, although the Commercial/Medicare Marketscan population is often considered to be generally representative of the US commercially insured population, the Medicaid population is only drawn from a selection of states and its representativity is uncertain. It is for that reason that any comparisons between these two populations can only be considered suggestive. Third, differences in the results obtained in the Commercial/Medicare and the Medicaid databases may come, at least in part, from differences in distribution of age, differences in the ethnicity or socio-economic status in the population enrolled in the databases, or differences in disease incidence among patients drawn from these diverse populations. Thus, although we found that the percentage of VPD-related admissions are higher and outcomes worse in the Medicaid population than in those with commercial or Medicare coverage (Figs. 1, 4), it is not possible—absent incidence data from the overall populations these cohorts were drawn from—to determine how meaningful these apparent differences are. If it is correct that the burden of VPD in older adults may disproportionately affect people in lower socio-economic groups (since access to Medicaid is, in most cases, based on household income relative to the poverty rate, it is sometimes used as a proxy for socio-economic status), prevention of these cases by vaccination, could lead to equity gains—something which is not often captured in standard cost-effectiveness analysis of vaccination [42]. Lastly, vaccination status of the hospitalized patients was not assessed which greatly limits insight into how much of the burden of disease described in this analysis is potentially preventable by vaccination. Inferring vaccination status from medical claims data is a challenge as vaccination status in this heterogeneous population is affected by multiple factors such as insurance type (e.g., commercial vs Medicare, degree of reimbursement versus out of pocket costs, etc.) and the long booster intervals for some vaccines that require tracing the history of patients over multiple years to assess coverage. Based on the insights of this analysis we recommend conducting in-depth subgroup analysis per vaccination type and associated diseases to investigate the impact of vaccination status in future studies.

However, even taking these limitations into account, we believe that this study shows that insurance claims data can be a productive source of information for these analyses. Given the potential impact of downstream effects of infections with—for example—influenza or herpes zoster, which are major contributors to poor health in older adults, not just for cardiovascular and cerebrovascular disease, but also for dementia, renal disease, and other conditions [17, 24]—understanding the impact of these infections on caseload, cost of treatment and outcomes is essential to a rational assessment of the importance of downstream effects of infectious disease and for designing the most efficient use of healthcare resources. More detailed studies on the impact of VPDs on healthcare resource use and inpatient costs (in particular, analyzing the relationship between admission data and community incidence of VPD) are urgently needed.

## Conclusions

The data presented in this paper suggest that VPDs in ageing adults may contribute more towards health outcomes and healthcare utilization than is usually recognized, and that medical insurance claims data on principal and secondary diagnoses are a potentially useful source of information for dissecting these impacts. This is based on the observation that the number of patients admitted to hospital with a principal diagnosis other than VPD who appear to also have concurrent VPD infections is greater than those with a principal diagnosis of VPD over the 3-year period studied. Excluding the principal diagnosis Certain infectious and parasitic diseases (ICD-10 Code range A00-B99) where infection of some kind was already diagnosed at admission, the two commonest principal diagnoses associated with a secondary diagnosis of VPD were Diseases of the respiratory system (ICD-10 code range J00–J99) and Diseases of the circulatory system (ICD-10 code range I00–I99) consistent with previous findings from population level studies of a potential causative role for VPDs in these illnesses.

## Supplementary Information

Below is the link to the electronic supplementary material.

Figure Sup 1. Distribution of the principal diagnosis at admission, expressed as a percentage of all claims registered for patients 50 years of age or older with the MarketScan Commercial Claims and Encounters (Commercial), and Medicare Supplemental (Medicare) databases (pooled data, n= 1,964,984) from July 1, 2016 to June 30, 2019. Principal diagnoses were sorted from most to least frequent, using the specific ICD-10 codes registered for the claim. I00-J99: Diseases of the circulatory system; M00-M99: Diseases of the musculoskeletal system and connective tissue; K00-K95: Diseases of the digestive system; S00-T88: Injury, poisoning and certain other consequences of external causes; A00-B99: Certain infections and parasitic diseases; J00-J99: Diseases of the respiratory system; C00-D49: Neoplasma; N00-N99: diseases of the genitourinary system; F01-F99: Mental, behavioural and neurodevelopment disorders; E00-E89: Endocrine, nutritional and metabolic diseases; G00-G99: Diseases of the nervous system; VPD, vaccine-preventable diseases; R00-R99: Symptoms, signs and abnormal clinical and laboratory findings, not elsewhere classified; G00-G89: Diseases of the nervous system; L00-L99: Diseases of the skin and subcutaneous tissue; Z00-Z99: Factors influencing health status and contact with health services; D50-D89: Diseases of the blood and blood-forming organs and certain disorders involving the immune mechanism; Q00-Q99: Congenital malformation, deformations and chromosomal abnormalities; H60-H95: Diseases of the ear and mastoid process; H00-H59: Diseases of the eye and adnexa; O00-O94: Pregancy, childbirth and puerperium; P00-P96: Certain conditions originating in the perinatal period; V00-Y99: External causes of morbidity (TIF 2058 kb)

Figure Sup 2. Distribution of the principal diagnosis at admission among patients who also had a secondary diagnosis of VPD, expressed as a percentage of all claims registered for patients 50 years of age or older with the MarketScan Commercial Claims and Encounters (Commercial), and Medicare Supplemental (Medicare) databases (pooled data, n= 1,964,984) from July 1, 2016 to June 30, 2019. Principal diagnoses were sorted from most to least frequent, using the specific ICD-10 codes registered for the claim. Abbreviations: VPD, vaccine-preventable diseases; A00-B99: Certain infections and parasitic diseases; J00-J99: Diseases of the respiratory system; I00-J99: Diseases of the circulatory system; S00-T88: Injury, poisoning and certain other consequences of external causes; K00-K95: Diseases of the digestive system; C00-D49: Neoplasma; N00-N99: diseases of the genitourinary system; R00-R99: Symptoms, signs and abnormal clinical and laboratory findings, not elsewhere classified; E00-E89: Endocrine, nutritional and metabolic diseases; G00-G99: Diseases of the nervous system; M00-M99: Diseases of the musculoskeletal system and connective tissue; D50-D89: Diseases of the blood and blood-forming organs and certain disorders involving the immune mechanism; L00-L99: Diseases of the skin and subcutaneous tissue; Z00-Z99: Factors influencing health status and contact with health services; F01-F99: Mental, behavioural and neurodevelopment disorders; H00-H59: Diseases of the eye and adnexa; H60-H95: Diseases of the ear and mastoid process; Q00-Q99: Congenital malformation, deformations and chromosomal abnormalities; O00-O94: Pregancy, childbirth and puerperium (TIF 2163 kb)
