# Supplementary figures and images for: Capturing the value of vaccination: impact of vaccine-preventable disease on hospitalization
Source: Aging Clin Exp Res. 2022 May 28;34(7):1551–61. doi: 10.1007/s40520-022-02110-2 (PMC9142834; doi:10.1007/s40520-022-02110-2)

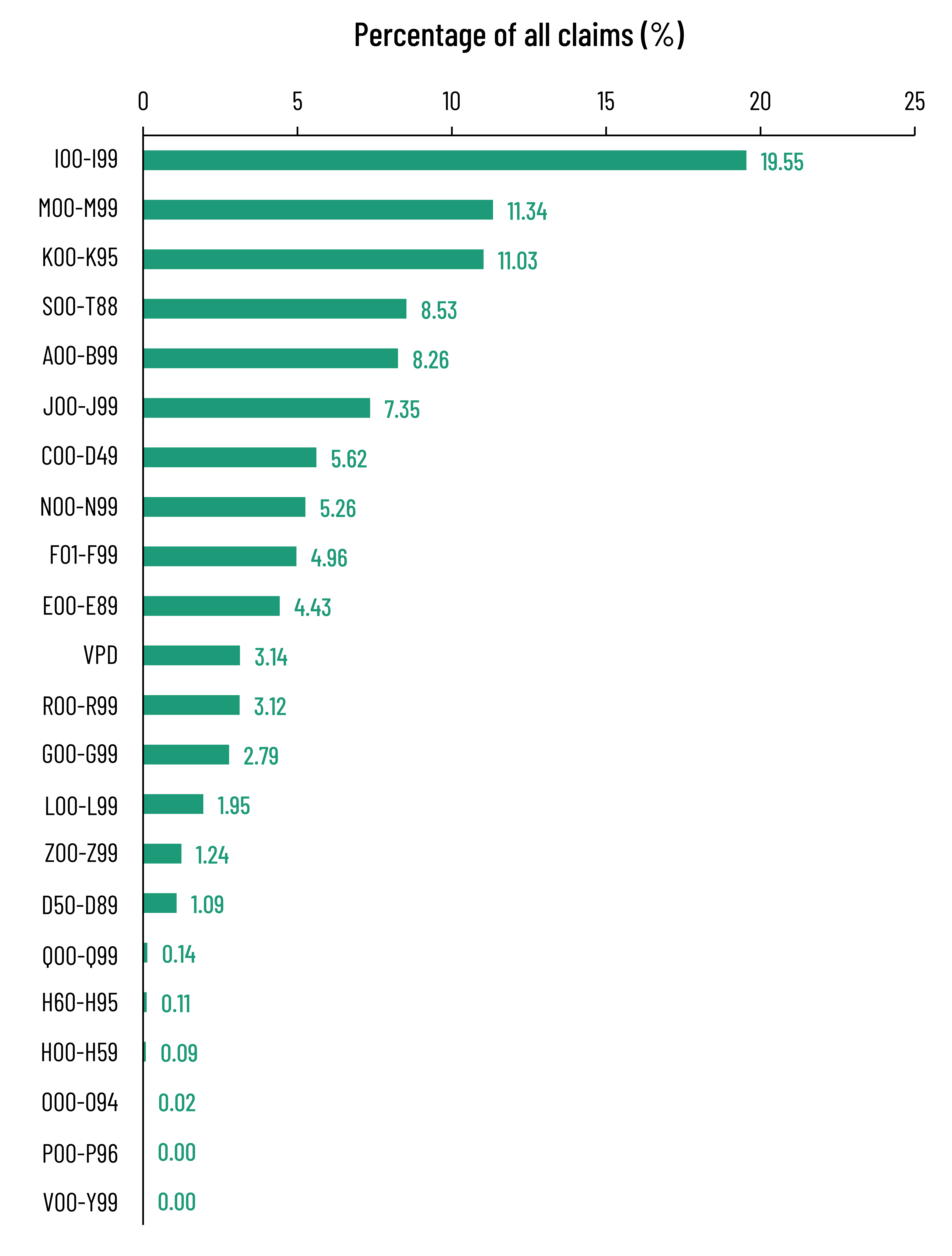

Supplement: Supplementary file 1 — Figure Sup 1. Distribution of the principal diagnosis at admission, expressed as a percentage of all claims registered for patients 50 years of age or older with the MarketScan Commercial Claims and Encounters (Commercial), and Medicare Supplemental (Medicare) databases (pooled data, n= 1,964,984) from July 1, 2016 to June 30, 2019. Principal diagnoses were sorted from most to least frequent, using the specific ICD-10 codes registered for the claim. I00-J99: Diseases of the circulatory system; M00-M99: Diseases of the musculoskeletal system and connective tissue; K00-K95: Diseases of the digestive system; S00-T88: Injury, poisoning and certain other consequences of external causes; A00-B99: Certain infections and parasitic diseases; J00-J99: Diseases of the respiratory system; C00-D49: Neoplasma; N00-N99: diseases of the genitourinary system; F01-F99: Mental, behavioural and neurodevelopment disorders; E00-E89: Endocrine, nutritional and metabolic diseases; G00-G99: Diseases of the nervous system; VPD, vaccine-preventable diseases; R00-R99: Symptoms, signs and abnormal clinical and laboratory findings, not elsewhere classified; G00-G89: Diseases of the nervous system; L00-L99: Diseases of the skin and subcutaneous tissue; Z00-Z99: Factors influencing health status and contact with health services; D50-D89: Diseases of the blood and blood-forming organs and certain disorders involving the immune mechanism; Q00-Q99: Congenital malformation, deformations and chromosomal abnormalities; H60-H95: Diseases of the ear and mastoid process; H00-H59: Diseases of the eye and adnexa; O00-O94: Pregancy, childbirth and puerperium; P00-P96: Certain conditions originating in the perinatal period; V00-Y99: External causes of morbidity (TIF 2058 kb) [file 40520_2022_2110_MOESM1_ESM.tif]

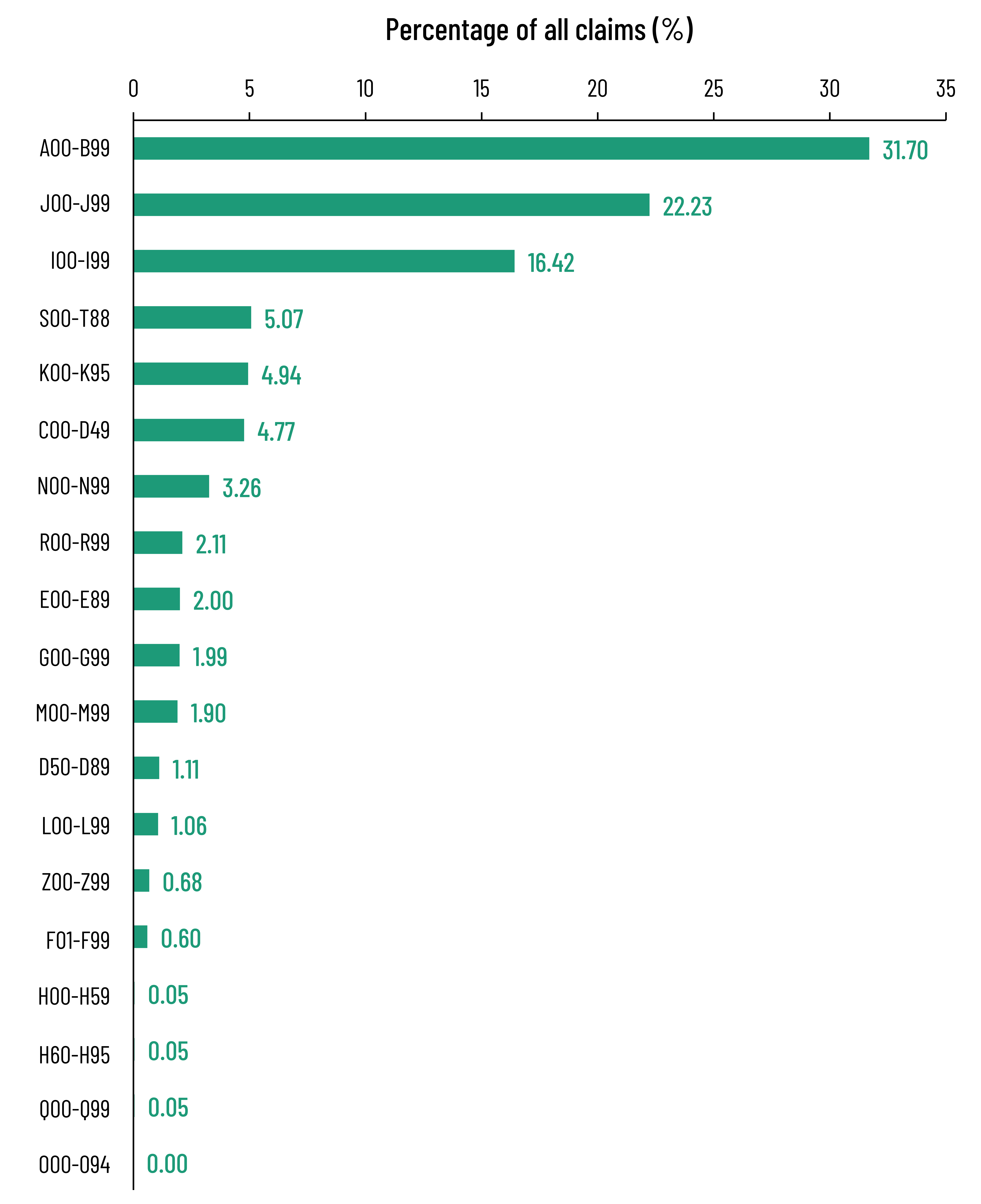

Supplement: Supplementary file 2 — Figure Sup 2. Distribution of the principal diagnosis at admission among patients who also had a secondary diagnosis of VPD, expressed as a percentage of all claims registered for patients 50 years of age or older with the MarketScan Commercial Claims and Encounters (Commercial), and Medicare Supplemental (Medicare) databases (pooled data, n= 1,964,984) from July 1, 2016 to June 30, 2019. Principal diagnoses were sorted from most to least frequent, using the specific ICD-10 codes registered for the claim. Abbreviations: VPD, vaccine-preventable diseases; A00-B99: Certain infections and parasitic diseases; J00-J99: Diseases of the respiratory system; I00-J99: Diseases of the circulatory system; S00-T88: Injury, poisoning and certain other consequences of external causes; K00-K95: Diseases of the digestive system; C00-D49: Neoplasma; N00-N99: diseases of the genitourinary system; R00-R99: Symptoms, signs and abnormal clinical and laboratory findings, not elsewhere classified; E00-E89: Endocrine, nutritional and metabolic diseases; G00-G99: Diseases of the nervous system; M00-M99: Diseases of the musculoskeletal system and connective tissue; D50-D89: Diseases of the blood and blood-forming organs and certain disorders involving the immune mechanism; L00-L99: Diseases of the skin and subcutaneous tissue; Z00-Z99: Factors influencing health status and contact with health services; F01-F99: Mental, behavioural and neurodevelopment disorders; H00-H59: Diseases of the eye and adnexa; H60-H95: Diseases of the ear and mastoid process; Q00-Q99: Congenital malformation, deformations and chromosomal abnormalities; O00-O94: Pregancy, childbirth and puerperium (TIF 2163 kb) [file 40520_2022_2110_MOESM2_ESM.tif]
